# 4263 The cardioprotective effects of ramipril during the course of experimental hypercholesterolemia in rabbits

**DOI:** 10.1017/cts.2020.96

**Published:** 2020-07-29

**Authors:** Zvezdana Zivorad Kojic

**Affiliations:** 1 University of Belgrade, School of Medicine

## Abstract

OBJECTIVES/GOALS: High cholesterol is among the major causes of cardiometabolic complications. People with high cholesterol have about twice the risk of heart disease as people with lower levels. Approximately every 40 seconds, an American will have a heart attack. Costs related to Heart Attack exceed 60 Billion USA dollars per year. Renin-Angiotensin-Aldosteron System (RAAS) is implicated in the genesis of coronary heart disease and in the perpetuation of heart failure. Angiotensin-Converting Enzyme inhibitors (ACE-I) have emerged as the treatment of choice for patients with all degrees of heart failure. Many clinical trials (Consensus, 1987; Save 1990) provide the evidence that ACE-I preserves cardiac function, prevents cardiovascular death, myocardial infarction & stroke and limit remodeling after myocardial infarction. However, there are still controversies in cardiology and a debate over cardioprotection is continuing:

- Do ACE Inhibitors have unique properties, beyond their antihypertensive effect?
- Can we protect the heart during hypercholesterolemia?
- In which way hypercholesterolemia affects mitochondria bioenergetics?
- How does ramipril affect mitochondrial bioenergetics during the course of experimental hypercholesterolemia?

Objectives/Goals were: To evaluate the mitochondrial actions of chronically administered ramipril (non-SH-containing ACE inhibitor) in cholesterol-fed rabbits by determining the influence of ramipril on:

- myocardial oxygen consumption (State 4, State 3), Respiratory Control Ratio (RCR), and adenosine diphosphate - oxygen index (ADP/O) and
- oxidative stress biomarkers

METHODS/STUDY POPULATION: ***Animal treatments***. In the course of twelve-week the male Chinchilla rabbits (n = 10/group) received once a day a single dose, in group: A - sunflower oil (control animals); B – atherogenic 2% hypercholesterolemic diet; C - atherogenic diet and ramipril (1 mg/kg) and D - ramipril (1 mg/kg) only. Animals were terminated in accordance with the U.K. “Animals (Scientific Procedures) Act.” ***Isolation of mitochondria** - Mitochondria from rabbit heart were isolated by tissue digestion (trypsin), fractionation and differential centrifugation. ***Mitochondrial respiratory functional measures*** (State 4 - Basal; State 3 - ADP-stimulated respiration, RCR and ADP/O) and biochemical markers of oxidative damage (the nitrite level) were measured polarographically (Clark electrode, YSI, USA) and spectrophotometrically, respectively, in isolated heart mitochondrial suspensions. ***Statistics*** - All results are reported as means ± SD. Comparisons between ramipril treated and control animals were performed by unpaired *t*-test or one-way ANOVA with a Tukey adjustment for multiple comparisons. A *P* value < 0.05 was considered significant for all tests. RESULTS/ANTICIPATED RESULTS: ***Plasma cholesterol levels:*** After a period of 12 weeks*

- Plasma cholesterol levels in control rabbits (A) were low (1.36 ± 0.23 mmol/l).
- Cholesterol-fed rabbits became hypercholesterolemic and their plasma total cholesterol level was higher even than 10 mmol/l. The level of total cholesterol in the high-cholesterol-diet group was significantly increased compared with the level in the normal-diet group (p < 0.01).
- In the high-cholesterol-diet group treated with ramipril (C), the plasma cholesterol level was not affected by the drug ramipril (10.54 ± 1.31 mmol/l). ACE-I ramipril did not infuence the concentration of total cholesterol.
- Plasma cholesterol levels in group D were low (1.46 ± 0.29 mmol/l).

***Mitochondria protein concentration:*** 10-15 mg per heart (0.5 mg/ml). ***Mitochondrial actions of ACE-I ramipril:*** a typical mitochondrial oxygen consumption rates in State 4 (basal) and State 3 (+ADP) in malate-energized heart mitochondria after 12-week treatment protocol highlights that, comparing to the control group:

- Atherogenic 2% cholesterol diet (B) caused a decline in mitochondrial function (in both, State 3 and 4) (−25%).
- Mitochondria from group C animals (treatment with ramipril along with 2% cholesterol diet) exhibited higher State 3 respiratory rates compared with group B.
- Mild inhibition of mitochondrial respiration was recorded in group D, in both respiratory states (V4&V3).

No significant difference between the groups were found regarding RCR. However, high significant statistical decrease (p < 0.01) was found in group B regarding ADP/O ratio. ***Nitrite levels:*** Rabbits receiving a supplementation of 2% cholesterol for 12 weeks (B) showed an increase in nitrite levels (115 ± 21 nmol/l vs 50 ± 9 nmol/l controls). However, It was attenuated by ramipril (C) (85 ± 16 nmol/l). Strong correlations were found between State 3 respiratory rate and the nitrite level (r = −.967, p < 0.05). It means: the higher level of nitrite – the lower oxygen consumption rate. DISCUSSION/SIGNIFICANCE OF IMPACT: The beneficial effect of ramipril was not noticed on plasma cholesterol levels. However, ramipril improves rabbit heart mitochondrial bioenergetics function during the course of experimental hypercholesterolemia.

- In cholestrerol-supplemented hearts myocardial oxygen consumption was markedly reduced (State 4 and State 3) compared to controls. Administration of high-cholesterol diet decreased not only the respiratory activity of rabbit heart mitochondria (RHM), but also the sensitivity of respiratory chain to ADP (ADP/O), while concomitantly caused an increase in nitrite production. Possible explanation: high-fat diet affects the fluidity of mitochondrial membrane – Electron transport chain (ETC) may be damaged, and unable to support high rates of respiration (e.g. substantial cytochrome c could be lost).
- Administration of ACE-I ramipril along with cholesterol diet partially abolish reduction in MQO2 and improved coupling efficiency (ADP/O). Possible explanation: reduced coupling efficiency means the coupling mechanism itself is altered (e.g. the respiratory complexes slip and pump fewer protons than normal and less ATP is produced per oxygen consumed. Ramipril partially improved coupling efficiency and increased the amount of ATP per oxygen consumed.
- RCR - No significant difference between the groups were found. High RCR indicates good function (a high capacity for substrate oxidation and ATP turnover). Low RCR usually indicates dysfunction. However, there is no absolute RCR value that is diagnostic of dysfunctional mitochondria, because values are substrate- and tissue-dependent.
- NO• exerts metabolic control over mitochondrial respiration. Group B: The lowered state 3-respiration in heart mitochondria seems to contribute to the increased NO production, and elevated nitrite level.
- In a system as complex as OXPHOS, conclusions about overall efficiency must involve measurements of: mito membrane potential, proton transport, ATP synthesis and modular kinetic analisys.

Measurements both, respiration & potentials will enable us to identify the primary site of effectors, allowing deeper quantitative analyses and better patients therapy.

